# Enablers and barriers to success among mothers planning to exclusively breastfeed for six months: a qualitative prospective cohort study in KwaZulu-Natal, South Africa

**DOI:** 10.1186/s13006-017-0135-8

**Published:** 2017-10-03

**Authors:** Ngcwalisa Amanda Jama, Aurene Wilford, Zandile Masango, Lyn Haskins, Anna Coutsoudis, Lenore Spies, Christiane Horwood

**Affiliations:** 1 Centre for Rural Health, University of KwaZulu-Natal, Durban, South Africa; 2 Department of Pediatrics & Child Health School of Clinical Medicine Nelson R Mandela School of Medicine, University of KwaZulu-Natal, Durban, South Africa; 3 Department of Health, Pietermaritzburg, KwaZulu-Natal South Africa

**Keywords:** Exclusive breastfeeding, Qualitative research, Self-efficacy, Child nutrition, Health workers, South Africa

## Abstract

**Background:**

Exclusive breastfeeding (EBF) for the first six months of life is the most important determinant of child health and development, and is the recommended feeding practice for all mothers. However, EBF rates remain low in South Africa. This study aimed to prospectively explore enablers or barriers to success among mothers who planned to exclusively breastfeed their infants for the first six months of life, in KwaZulu-Natal, South Africa.

**Methods:**

A qualitative, longitudinal cohort design was adopted. Women were recruited during pregnancy from the catchment area of two hospitals (one urban and one rural) and purposively sampled to include working women, teenagers, and HIV positive pregnant women. This analysis relates to 22 women, from 30 women recruited, who planned antenatally to exclusively breastfeed for six months. These mothers were interviewed monthly for six months postpartum. Infant feeding practices were explored at each visit using in-depth interviews and 24 h feeding recall assessment. Framework analysis was conducted for qualitative data, and quantitative data analyzed using descriptive statistics.

**Results:**

A total of 125 interviews were conducted between November 2015 and October 2016. Among 22 mothers who planned to exclusively breastfeed for six months, 17 reported adding other food or fluids before six months, and five reported exclusively breastfeeding successfully for the first six months. Key themes showed that all mothers relied strongly on health workers’ infant feeding advice and support. All mothers experienced challenges regardless of whether they succeeded in EBF, including inappropriate advice from health workers, maternal-baby issues, pressure from family members and returning to school and work. However, those who were successful at EBF for six months reported that high breastfeeding self-efficacy, HIV status and cultural meaning attached to breastfeeding were underlying factors for success.

**Conclusion:**

Health workers are key players in providing infant feeding information and support, yet some health workers give mothers infant feeding advice that is not supportive of EBF. Strategies to improve health workers’ competency in infant feeding counselling are needed to better prepare pregnant women to overcome common breastfeeding challenges and build mothers’ confidence and self-efficacy, thus increasing EBF rates.

**Electronic supplementary material:**

The online version of this article (10.1186/s13006-017-0135-8) contains supplementary material, which is available to authorized users.

## Background

There is consistent and robust evidence, globally, of the importance of breastfeeding, particularly exclusive breastfeeding (EBF), for improving child health and development and reducing infant mortality [1, 2]. EBF is defined as feeding breastmilk only and no other liquids or solids except vitamins, mineral supplements or prescribed medication [3], and is the recommended feeding practice for all babies before the age of six months. However, even with its potential and actual benefits, EBF rates remain well below the global target of 50% for six months [4]. EBF among South African mothers has increased in recent years, but is still well below global targets, with an estimated 31.6% of infants being exclusively breastfed for the first six months [5]. Despite high rates of breastfeeding initiation, early cessation of breastfeeding and mixed feeding before six months of age are common practices amongst mothers in South Africa (SA) [6]. The 2016 South Africa Demographic and Health survey showed that among children aged 0–5 months, 25.2% were not breastfed at all, 11.4% were receiving breastmilk and other milk, and 17.6% were being given complementary feeds [5].

The SA National Department of Health has highlighted a number of the factors that influence mothers’ decisions to exclusively breastfeed for the first six months: aggressive promotion of infant formula by manufacturers; challenges to breastfeeding in the workplace; teenage mothers leaving their babies to go back to school; lack of family and community support; poor involvement of men in supporting breastfeeding; and confusion about the risk of HIV transmission and breastfeeding [7]. In South Africa, HIV prevalence is high, with 29.7% of women attending government antenatal clinics testing HIV positive in 2013 [8]. Teenage pregnancy is also very common with a recent national household survey finding that 27.8% of women aged 19 years had started childbearing [5]. Access to services for pregnant women and children is free in government health facilities in SA, with very high coverage of routine antenatal care, facility-based delivery, and immunisation of infants [9].

In addition, in their conceptual framework, Rollins et al. emphasize that individual factors such as the mother’s subjective norms regarding infant feeding, previous breastfeeding experience, her age, knowledge, attitudes, beliefs, and expectations regarding breastfeeding, are all important determinants of successful breastfeeding [1]. Psychosocial factors such as mothers education and provision of adequate support after birth are significant determinants of initiation and sustaining of breastfeeding, influencing self-efficacy and mother’s confidence that she can successfully breastfeed [1]. This is supported by the theory of self-efficacy, which can be defined as a person’s belief in their ability to produce a level of performance in a particular activity, and can be influenced the persons experience of successfully achieving a particular goal, their observation of others doing so, as well as with verbal persuasion [10]. Breastfeeding is a behaviour that relates to the relationship between mother and baby, and perceptions that the baby is satisfied and content are strong determinants of breastfeeding success [1]. These findings highlight the complex nature of infant feeding practices and the ability to sustain infant feeding decisions.

A number of studies conducted on infant feeding choices and influences in SA have employed descriptive and quantitative cross-sectional methods [11–14] that tend to capture participants’ current feeding patterns and rely on participants’ memory. WHO has highlighted that adopting more prospective longitudinal methods is more valid in capturing the complexity of feeding patterns [15]. In this study, a qualitative methodology using in-depth interviews conducted prospectively over six months, which provided an appropriate methodology for contemporaneous exploration of the complex situations mothers faced while feeding their babies, and the factors influencing decision-making.

If the global target of 50% EBF for six months is to be achieved [4], effective contextualized interventions to promote breastfeeding are needed. This is particularly relevant in South Africa where society and health systems face complex challenges, including high levels of inequity, unemployment, poverty and gender-based violence, with low rates of EBF [16], and high infant mortality [5]. In-depth understanding of how mothers make feeding decisions is necessary to design such interventions. We report the findings of a longitudinal cohort study, conducted among mothers who planned to exclusively breastfeed their infants for six months, and describe the factors that facilitated or acted as barriers to achieving their goal.

## Methods

### Study design

The study adopted a longitudinal qualitative design to explore individual narratives on infant feeding choices and practices from birth to six months of life, in order to prospectively capture critical moments and processes that were involved in participants’ infant feeding choices during this period [17]. The framework method was selected for data analysis because this was aligned with the research question and the study aims. The framework method is popular for its flexibility, in that it is not linked to a particular theoretical approach, but can be modified to fit many qualitative approaches that aim to generate themes [18].

### Study site

The study was carried out in two sites in KwaZulu-Natal (KZN), a deep rural site in northern KZN and an urban area in Durban. Sites were selected in partnership with the KZN Department of Health, based on whether the local hospital had a lactation advisor on site. The rural area has a population of 120,000, characterized by high illiteracy rates, widespread poverty, and poor service delivery, with people surviving through migrant labour, subsistence farming and government grants, often shared amongst entire households. This area has one hospital for a population burdened with HIV, tuberculosis and malnutrition. The urban area in Durban houses approximately 290,000 residents in largely formal settlements with better levels of service delivery. The area is also affected by HIV but with lower rates of malnutrition [19].

In all areas of KZN, including the two study areas, a high proportion of women (> 90%) deliver inside a health facility, most commonly at the local district hospital [9]. KZN Department of Health policy recommends that mothers and babies be discharged four hours after a normal delivery but they frequently stay longer for practical reasons. The hospitals in both study areas have a lactation advisor, who is a lay counsellor trained to support initiation of breastfeeding in the postnatal ward. The rural and urban clinics had 22 and 18 CHWs respectively, deployed to the local area. CHWs are expected to visit mothers at home during the antenatal and postnatal periods.

### Sampling and recruitment

**Fig. 1:**
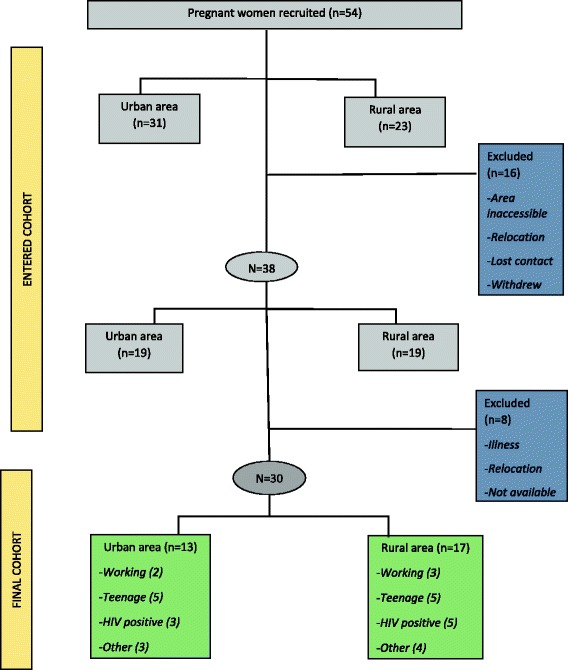
Participants’ sampling and cohort profile

Women aged 15 years or older who were more than 36 weeks pregnant, were approached for recruitment in antenatal clinics at each site by trained field workers. Study recruitment employed a purposive sampling technique to ensure that participants included teenagers (aged 15–19 years), working women and HIV infected women. These particular groups were selected to ensure that perspectives of groups who are known to have different breastfeeding practices were included in the sample [20, 21]. Participants who did not plan to reside in the area with the baby for the first six months after delivery were excluded from the study. Eligible women were given a brief overview of the study and they provided contact details. Fieldworkers then contacted them to arrange a home visit, where recruitment was completed and informed consent obtained. Figure 1 shows the recruitment process and the cohort profile.

### Data collection

Data collection was conducted in participants’ households by two fieldworkers, one in each site, who had received two weeks of intensive training. Field workers were recruited in a competitive selection process, both had a high school education and some experience with data collection. In the urban site the field worker resigned after three months and data collection was completed by a research intern with a degree in research psychology. Field workers were not from the areas where the study was conducted, and were not known to participants.

Field worker training covered research ethics, obtaining informed consent, and data collection, with the strongest focus being on developing qualitative interview skills. Fieldworkers underwent extensive practice conducting interviews using role-plays and during piloting. The quality of the interviews was reviewed by experienced qualitative researchers, and feedback provided to the field workers until they were assessed as being competent to undertake all research activities to a high quality. During data collection, researchers listened to interviews on an ongoing basis to monitor quality of interview skills, and feedback given to fieldworkers as necessary.

During the enrolment visit conducted during the antenatal period, baseline data was collected using a structured quantitative questionnaire, which included information about participants’ socioeconomic characteristics and her infant feeding plans. Mothers were requested to state whether they planned to feed their baby breastmilk only, formula milk only, or both breast and formula milk. At subsequent visits an in-depth interview guide was used to explore three main topics; current feeding practices; reasons for adopting particular feeding practices; people and situations that influenced infant feeding decisions; contacts with the health service. Two interview guides were developed by the study team, and were piloted with mothers of young children attending a local clinic and amended accordingly. The interview guide for the first postnatal visit included information about experiences after delivery, but was otherwise similar (see Additional files 1 and 2). Mothers also answered a quantitative 24 h food and fluid recall questionnaire at the beginning of each visit, this was based on the WHO tool [15] and translated into Zulu.

#### Procedure

Seven visits were conducted per participant in their home. At the enrolment visit written informed consent was obtained from all participants for the entire six month study period. Among young teenagers (age 15–17 years) consent was obtained from parents or legal guardians and assent from the participant. Participants provided the expected date of delivery of their baby and were contacted regularly by field workers until the baby was born. After which, arrangements were made to visit the household to conduct the first interview with the mother, within the first two weeks after birth.

Monthly face-to-face in-depth interviews were conducted in the participants’ language of preference, either IsiZulu, IsiXhosa or English. Interviews were audio recorded. Fieldworkers began each interview with a review of the content of previous interviews, thus allowing fieldworkers and participants to keep the focus on longitudinal elements of infant feeding practices and decision-making [17].

### Ethical considerations

Ethics clearance for this study was obtained from the Biomedical Research Ethics Committee at the University of KwaZulu-Natal (BE301/15) and the KwaZulu-Natal Department of Health. To preserve anonymity, codes were assigned to each participant based on the area, the category and number of the visit. Audio recordings and transcripts were stored at UKZN in a password protected file.

### Data analysis

All interviews identified were transcribed verbatim and translated into English. Framework analysis was used to analyse the interview data. The framework method was appropriate for this study as it provided a systematic and explicit approach to categorizing and organising this large qualitative dataset [18]. Analysis was based on pre-determined research themes (drawn from the interview guide) as well as inductive themes that emerged from the interview data. Framework analysis was selected as particularly appropriate for a longitudinal study because it allowed researchers to compare and contrast data within cases and across cases.

Framework analysis comprised five stages, beginning with a process of familiarization with the transcripts to gain an overview of the content. This was followed by the development of an analytical framework based on identified research questions as well as on themes that emerged. This framework was then applied to the individual transcripts and data charted into categories based on these identified themes. Finally, a process of mapping and interpretation was undertaken [22].

Data was exported to NVivo v11 for analysis and two skilled qualitative researchers performed inter-coder reliability to ensure analysis consistency across all transcripts.

Quantitative data gathered was double entered into EpiData v3.1 and descriptive analysis was conducted using SPSS v23 to generate frequencies on participants’ demographic information, feeding plans, and infant feeding patterns over six months. We defined exclusive breastfeeding according to the WHO definition [3], which states that the infant receives only breast milk from his or her mother or expressed breast milk, and no other liquids or solids with the exception of drops or syrups consisting of vitamins, mineral supplements, or medicines.

## Results

**Fig. 2:**
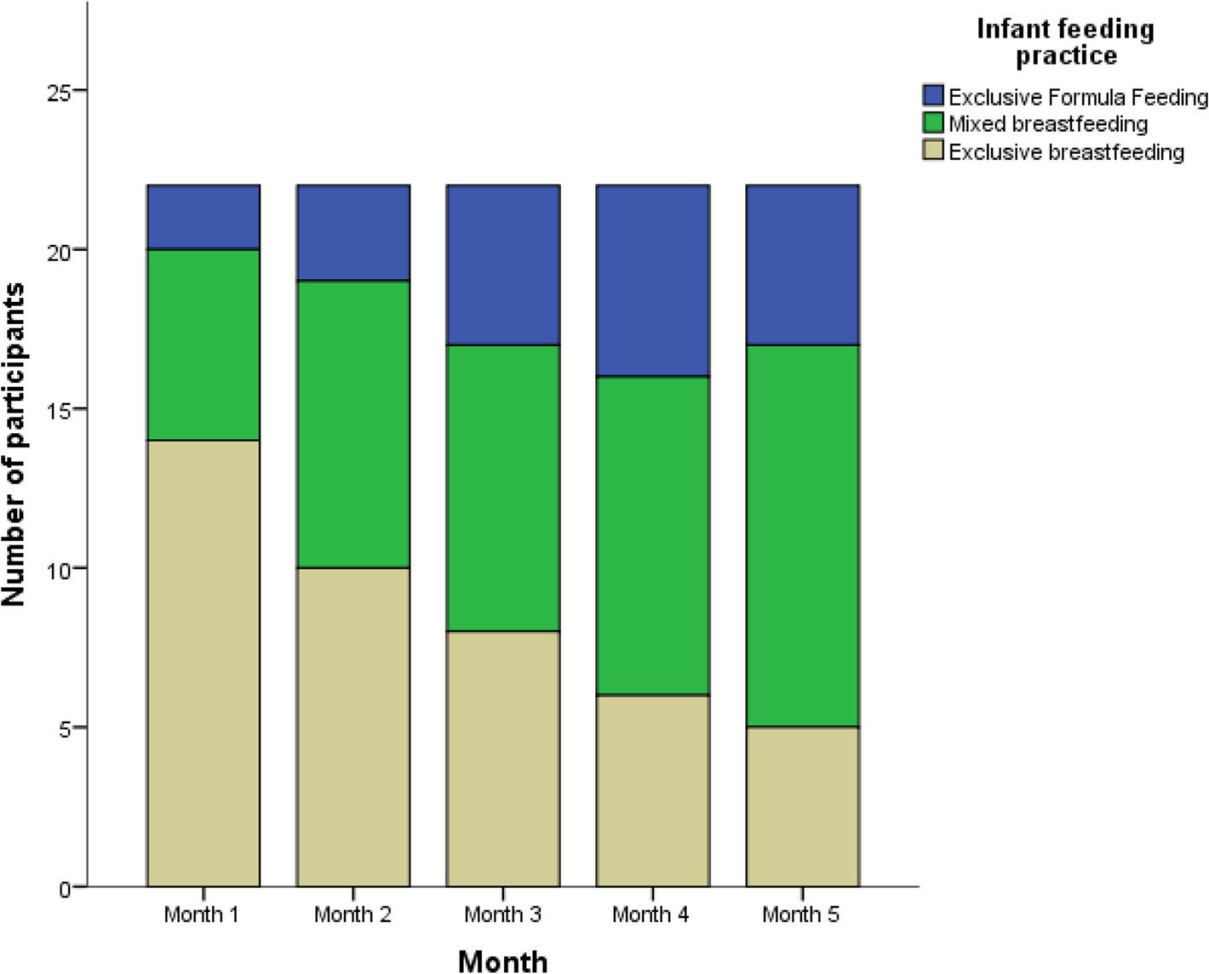
Participants’ reported feeding practices from month one to month five from the 24 h food and fluid recall

These results relate to 22 participants who stated their plans to exclusively breastfeed for six months during the enrolment visit conducted prior to delivery. A total of 125 in-depth interviews were conducted between November 2015 and October 2016. The median age of participating mothers was 25.5 years (IQR 18–34). Participants’ socio-demographic details are shown in Table 1. Figure 2 shows infant feeding patterns of participants from month one to month five from the 24 h food and fluid recall data, the last visit is excluded because this was after six months of age and mothers would not be expected to exclusively breastfeed. Of 22 mothers, 17 (nine rural; seven urban) gave their babies other liquids and/or solids before they reached six months, and five (three rural; two urban) successfully exclusively breastfed for six months. Among the five mothers who exclusively breastfed for the full six months, three were HIV positive, one was a fulltime working mother and one was a stay-at-home mother from Zimbabwe. Mothers who mixed fed added other food and fluids at different time points (Fig. 2).

**Table 1:** Socio-demographic details of mothers who planned to exclusively breastfeed

*N* = 22	*n*	*%*
AGE CATEGORY		
15–19	7	31.8
20–26	6	27.3
27>	9	40.9
EDUCATION- Highest grade passed		
Grades 4–7/Primary school education	1	4.5
Grades 8–11/Secondary school education	14	63.6
Grade 12/Matriculated	7	31.8
Mother is in a relationship with the child’s father	20	90.9
Mother is staying in same house as the child’s father	12	54.5
WORK		
Current paid work	3	13.6
ADDITIONAL INCOME SOURCES		
Mother receives money from employer	2	9.1
Mother receives money from self-employment	2	9.1
Mother receives child support grant for any child	8	36.4
Mother receives disability grant	2	9.1
Mother receives maintenance/money from partner	16	72.7
Mother receives money from family members	8	36.4
Other sources	2	9.1
Feeding practices with previous child (first 3 months)		
Breast milk only	11	50.0
Formula only	1	4.5
Breast milk and formul	2	9.1
No previous children	8	36.4
ACCESS TO SERVICES		
No. of antenatal visits attended during current pregnancy		
< 4 antenatal clinic visits	6	27.3
4–8 antenatal clinic visits	16	72.7
HIV testing- mother reports ever tested for HIV	22	100
Mother taking antiretroviral treatment	8	36.4
A community health worker visits the household	4	18.1
Visited by a community health worker during this pregnancy	2	9.1

### Barriers to exclusive breastfeeding for six months

Barriers to exclusive breastfeeding were similar among all participating mothers, and comprised of interplay of health system factors, maternal-baby factors and social factors (family pressure and returning to work/school). However, although multiple factors may have played a part, for each mother who added other food or fluids before six months a single precipitating factor was identified that directly led to cessation of EBF. Table 2 shows the main reason for adding other food/fluids for each of the 17 mothers who failed to EBF for six months.

**Table 2:** Summary of the main precipitating factors for adding other food or fluids among those mothers who failed to EBF for 6 months

Mother ID	Health system	Mother-baby factors	Family pressure	Returning to Work/School
W03: Urban, HIV positive	Advised by the nurse to stop breastfeeding at *three months* upon returning to work			
M05: Rural, Other			Pressured by aunt to give traditional medicine at *one month*	
M09: Rural, HIV positive		Mother perceived milk insufficiency at *five months*		
W10: Urban, Teenager	Advised by the nurse to give water within *one month*			
M13: Rural, Teenager				Introduced formula milk upon returning to school *at two months*
M14: Rural, Working				Baby given water with sugar while mother was at work *at three months*
M15: Rural, Teenager			Pressured by the grandmother to introduce complementary foods at *two months*	
W15: Urban, Other	Advised by the nurse to give water at *three months*			
W17, Urban, Teenage		Mother perceived milk insufficiency at *four months*		
W19: Urban, Teenager	Baby given pre-lacteal feeds at the hospital within *one week*			
M19: Rural, Other		Baby given water because breastmilk not coming out during the first *three days*		
M22: Rural, Teenager			Instructed by her mother (child’s grandmother) to feed the baby soft solids at *four months*	
M24: Rural, HIV positive				Mother struggled to express at work and introduced formula milk *at three months*
W30: Urban, Teenager		Baby struggled to latch and was given formula milk at *one month*		
W31: Urban, Working	Baby given pre-lacteal feeds at the hospital within * one week*			
W47: Urban, HIV positive		Baby not getting full from breast alone at *five months*		
W49: Urban, Other			Advised by her mother to add porridge at *three months*	
Total no of mothers	5	5	4	3

### The health system

Most participants in the study attended ANC visits more than four times (Table 1), and all delivered their babies in hospital. Two participants received a home visit from community health workers during the antenatal period. During ANC visits, all participants received health education about the benefits of breastfeeding. All mothers had a general understanding based on the ANC education, that breastfeeding is more nutritious than any other milk and protects infants from getting diseases. One participant from the rural site revealed the knowledge she received from the ANC.

*P: The sisters (nurses) are the one who usually advise us, they teach us in the morning after prayer. One day we learnt about breastfeeding, on why it is important for a woman to breastfeed. She explained everything and others were answering questions, and I got some knowledge that I didn’t know about, and the sister complimented breastfeeding. She taught us that breast milk is important for the baby and the mother as well. She first said it creates love between the mother and the baby. I think I know some few points, if I’m not lying, that the baby…ok first they say the baby becomes smart, he grows well, his poop doesn’t smell bad, he’s protected from diseases such as flu when he’s breastfeeding (M04_HIV pos_month 3).*

Once participants had given birth, they reported that they were assisted with initiation of breastfeeding by health workers at the hospital. One teenage mother narrated:

*P: Yes, there is a nurse who was supervising me whether I feed him right, is he sucking ok? If there are the sounds she will tell me that he’s not sucking well, I must take my breast out and put it back again until he feeds quietly (W10_Teen_month 01).*

In one of the two sites (urban hospital) it was common practice in the hospital for newborns to be given pre-lacteal feeds while mothers waited for their milk to come in or were recovering post caesarean section. This was a barrier to EBF for two mothers, because they were no longer designated as EBF according to the strict definition used. However, only one mother perceived this as a barrier to breastfeeding and both mothers resumed breastfeeding after returning home. One first-time mother described this:

*P: I even told the nurse I don’t have milk and then the nurse gave my baby formula (W31, working _month 01).*

When mothers experienced breastfeeding challenges at home, they usually consulted with the health workers. The data revealed that when mothers sought advice from the health workers, they were frequently given inappropriate advice that did not support EBF, and for many this was a key barrier to EBF and led directly to mothers failing to exclusively breastfeed.

Some mothers mentioned how they were encouraged by the health workers to give the baby water after enquiring about whether the baby needs water or reporting constipation concerns, suggestions which they implemented. A new teen mother mentioned:

*P: The last time he poo was on the day when he was born, on the 29th. Two days after he still didn’t, so I gave him water because they said at the clinic I must give him water with sugar and if it continues I must go to the chemist and get the medicine (W10, teen_month 01).*

For another mother, water was suggested by a health worker to help keep the baby hydrated.

*P: When I went back to the sister (nurse) to ask about whether I should give the baby water she said “just make sure you boil it”.*

*I: And how old was your baby when you received this message if you can remember?*

*P: Between 10 weeks and 14 weeks. They said you can give him 5 spoons a day to avoid dehydration and that time it was very hot. After that consultation, I continued with water (W15, other_month 03).*

An HIV positive working women expressed that she stopped breastfeeding immediately after returning to work as encouraged by the health worker:

*P: She (the baby) stopped breastfeeding because of the reasons I’ve stated before, that I am scared that the nanny/aunt will give her water, (or) medicine which is wrong. So I discussed with the nurse on our last visit, which was on the 29th, because I decided to go back to work on the 1st. I asked her to advise me since I’m deciding to stop breastfeeding, she said to be on the safe side she is encouraging me to stop breastfeeding (W03_HIV pos_month 03).*

### Maternal-baby factors

All mothers reported success in initiating breastfeeding before being discharged from the hospital. However, after returning home, some mothers experienced breastfeeding as tiring, embarrassing, and not enough to sustain the baby’s hunger.

The most commonly cited barrier to exclusive breastfeeding was the perceived lack of breast milk to sustain the baby. The reasons for this perception included the baby constantly crying, baby wanting to breastfeed for longer, or no milk being produced when expressing. This ‘forced’ mothers to add other solids or introduce formula milk even when the advice from the clinic was to only give breast milk:

*P: I feed him because there is nothing else I can do when the baby is hungry, he’s hungry I’m forced to give him porridge because if I continue to listen to the people from hospital and clinic the baby will go hungry (M13, other_month 05).*

For some mothers, especially HIV positive mothers, the perceived lack of breast milk led them to stop breastfeeding altogether.

*P: The milk wasn’t coming out of my breasts and the baby was crying, even when I was expressing nothing came out so I thought it better to stop breastfeeding, instead of mixed feeding him. So I fed him formula only.*

*I: So how do you feel that you stopped him from breastfeeding before six months?*

*P: It didn’t feel right because I didn’t plan like that but I told myself that it’s better now because he’s getting full (M09, HIV pos_month 05)*.

Some mothers mentioned that when they started moving around, it became hard to breastfeed for longer periods as breastfeeding became tiring and inconvenient. This led to them introducing other foods in addition to breastmilk.

*P: I will stop him now because he’s breastfeeding a lot, although I have plenty, but no this person (baby) is breastfeeding a lot. I will now give him formula because it is too much for me (M22, teen_month 05).*

There were also a few cases where the mother reported that the baby did not want to breastfeed at all. For some mothers this happened soon after delivery where mothers mentioned that they believed this was due to having big or small nipples, resulting in baby struggling to latch on. In these cases, mothers used expressing breast milk as a strategy, which enabled them to continue to give breast milk exclusively.

*P: Yes, he doesn’t touch it, if I put my breast in his mouth he cries I don’t know why he does that. So, I express my breast milk into his bottle and that is when he feeds (M14, working_month 02).*

For some mothers, latching on was a barrier and led to the mother introducing formula milk.

*P: What made me to stop…actually I didn’t stop him. He was breastfeeding during the night, and during the day he feeds formula milk, but now he didn’t want breast milk, he’s used to feeding formula with a bottle. When I give him my breast he would just push it with his tongue and not want to latch on it so we stopped like that. I didn’t plan to stop him, he just stopped by himself (W10, teen_month 05).*

How mothers feel about breastfeeding in public places was another factor highlighted in this study, particularly among teenage mothers residing in the urban area, who chose to formula feed when in public places:

*I feed him bottled milk not anything because I don’t like to take out my breasts in public and feed the baby, I don’t like it. I can’t, but I do see some other people doing it, but no, I cannot imagine myself doing it (W19, teen_month 03).*

From this study we saw that those who were motivated to give the baby breast milk only, were comfortable breastfeeding even in public areas.

### Pressure from family to add other foods or liquids

All mothers, except one, stayed with their partners or family members. Mothers reported often being asked by significant others about when to add solid foods, and feeling pressured to do so.

*P: The baby’s father supports that I am giving the baby food, he’s happy because he was always asking me “when will the baby start eating” I told him “after six months he’ll start eating food” but he kept asking until I added food at 5 months (M05, other_month 05).*

Several mothers mentioned lack of support from family members, as being another barrier for mothers who wished to exclusively breastfeed for six months. An HIV positive mother described having added other foods as a result of pressure, a decision which her aunt supported.

*P: They said (her family) I must stop breastfeeding him and give him infant formula, maybe he’ll get full. They just said…they said our kids will die if we keep on listening to the nurses because you will find that their (nurses) kids are eating and ours are not (M08, HIV pos_month 02).*

The use of traditional medicine or practices was often mentioned by participants from rural areas. Although most mothers were against the use of traditional medicine, they were told by elders to use them. A young mother talked about how her child’s paternal aunt instructed her to give ‘their’ baby traditional medicine as a way of keeping up with the family’s tradition.

*His aunt told me that all of the children from their clan usually get traditional medicine to get better and remove all the dirt in the stomach, so they could be strong and better. They are the ones who showed me where to get it, they instructed me with everything, then I did it because it’s their baby, I must follow their rules (M05, other_month 02).*

Inability to reject opposing messages meant that some mothers changed their plans to exclusively breastfeed in response to family pressure. In particular, some teenage mothers being young and being expected to be obedient, were unable to go against those family members who suggested other feeding practices.

*I: When grandma tells you that to breastfeed only is wrong, how do you respond?*

*P: I stop it and do what she says (M01, teen_month 02).*

### Returning to work or school

Returning to school or work was a barrier to EBF for many mothers who introduced formula milk at that time and only breastfed when they were with the baby.

*P: Yes, I’m mixed feeding him because he eats formula when I’m not at home, during the day, and then when I come back he stops the bottle and breastfeeds (M13, teen_month 02).*

One fulltime working mother was committed to exclusive breastfeeding even while working, she expressed breast milk, and this enabled her to eexclusively breastfeed even when she was away from the baby. She described:

*Because when I’m at work I can express…oh I express 4 bottles and when I come back from work he still hasn’t finished them, everything is going well so far (M14, working_month 06).*

### Enablers among mothers who successfully exclusively breastfeed for six months

Self-efficacy, commitment, HIV status and cultural meaning attached to breastfeeding were found to be some of the underlying factors for mothers who successfully exclusively breastfeed. A mother from Zimbabwe stated how for her breastfeeding symbolized the meaning of motherhood:

*I have to do it…that is what being a mother is, to breastfeed, you know... and you have that bond. Me and my kids, that one (older child), we still have that bond (W15, other_month 01).*

When visited at six months, the same mother stated that her commitment and resolve was an enabling factor that helped her to successfully EBF. This mother also planned to stay with her baby, not returning to work, for six months in order to EBF for this period.

*P: You know with breastfeeding, it’s just commitment. If you tell yourself that you want to do this, you will do it no matter what people might say. Even if there are stumbling blocks along the way, so I think it is all about committing yourself (W15, other_ month 06).*

For HIV positive mothers, the motivation to EBF came from the fact that they did not want to infect the baby through mixed feeding. This resulted in their maintaining exclusive breastfeeding for six months regardless of challenges experienced along the way. The mother below narrates.

*P: I wanted to prove what the lactation advisor said at the hospital that…sometimes it (HIV) creates trouble, like when the baby is 5 years old he/she dies. Sometimes when you mix formula and breast milk the baby becomes (HIV) infected. I want to see if I can give birth to a baby who is negative while I am positive (W07, HIV pos_month 03).*

Another key enabling factor mentioned by mothers was the strong belief that breast milk was sufficient for the baby:

*P: Maybe the main reason is that my milk is still plenty otherwise if he was looking hungry or something maybe I was going to introduce something (W15, other_month 04).*

Those who were committed to exclusively breastfeeding appeared better able to stick with their decisions despite pressure and discouragement from friends and family. Such participants were most often HIV positive and older.

*P: When they talk I pretend as if I hear them but I don’t do what they are saying (M08, HIV pos_month 03).*

Another participant described why she rejected conflicting messages from others.

*P: No, I don’t listen to a person who is not well educated about this, I usually listen to people who are well educated because he/she (uneducated person) will tell me something that is not right. I usually listen to those that are educated and ask them on how should I breastfeed my baby and they explain to me. Because if I listen to other people, they won’t help me (M11, HIV pos_month 04).*

## Discussion

The importance of exclusive breastfeeding in improving children’s health is widely known, but poor breastfeeding rates are still reported in many developing countries, particularly in South Africa. This longitudinal prospective study sought to provide insights into the experiences among mothers who intended to exclusively breastfeed for six months in urban and rural settings in KZN. Our findings showed that multiple factors hindered mothers’ plans for six months EBF. However, for each mother a single precipitating factor was identified which led the mother to mixed feed. Health system factors and maternal-baby factors were the main precipitating reasons why mothers failed to exclusively breastfeed, followed by social factors (pressure from the family and returning to work or school). In contrast, mothers who were HIV positive and those who attached strong cultural meaning to breastfeeding, were motivated to exclusively breastfeed. This supports findings from previous research done in the similar setting, where women’s HIV status was found to influence exclusivity of breastfeeding [23].

Knowledge about the benefits of breastfeeding were conveyed by health workers during the routine ANC visits, where both HIV negative and HIV positive mothers were encouraged to exclusively breastfeed for six months. Most participants in our setting attended more than four ANC visits giving health workers extensive opportunities to encourage mothers to breastfeed and empower them with knowledge to refute opposing advice from family and friends. This study showed that infant feeding education was successful in encouraging mothers to choose breastfeeding, as over 70% of mothers in the study cohort chose to exclusively breastfeed their infants. Evidence from Nabulsi [24] emphasizes the importance of mothers’ knowledge about the benefits of breastfeeding as an underlying factor for maternal commitment to breastfeed.

Health workers continued being the source of advice for mothers, especially when mothers experienced challenges at the hospital with initiating breastfeeding and with maintaining EBF at home. Yet, advice from health workers was frequently not supportive of EBF and this advice was the main reason for stopping EBF for one third of mothers who did not successfully exclusively breastfeed. Health workers providing breastfeeding messages in South Africa may include lay health workers, enrolled nurses and registered nurses, who have all received training in breastfeeding. Inconsistent messages provided by health workers have been found in other settings [25], particularly in the setting of high HIV prevalence [26]. For South Africa, the confusion exists more in the context of HIV, with some health workers encouraging use of formula and others breast milk [27]. The belief about giving infants water is a tradition mostly reported amongst parents or family members [26, 28], however in our study health workers also encouraged this practice. Health workers in the urban hospital routinely provided pre-lacteal feeds, disrupting mothers’ plans to breastfeed and putting the infant at risk of illness [29, 30].

Our study suggests that advice given by health workers to mothers experiencing challenges with breastfeeding is one of the key determinants influencing mothers’ feeding practices [31]. Therefore, health workers not only have to provide clear counselling messages, they also have to be able to respond to challenges experienced by mothers. Health workers’ lack of ability to solve lactating mothers’ problems, poses a threat to sustained EBF and further skills training for health workers aimed at practical skills could form part of the solution. However, given the shortage of professional health workers, solutions could include greater involvement of the nutritional advisors in KZN [32], in the feeding counselling of mothers. Peer counselling has been shown to be effective in improving feeding practices in several low to middle income countries including South Africa [33, 34]. Further, research to explore health workers’ beliefs and understanding about the issue of giving water, infant feeding in the context of HIV, and other inappropriate practices could be conducted.

Most participants who managed to exclusively breastfeed for six months were HIV positive, suggesting that in this situation infant feeding messages from clinic health workers are in line with the current WHO guidelines [35]. In addition, family support was more common among HIV infected mothers who had disclosed their HIV status to significant others, which helped mothers to sustain EBF. Even so, the knowledge or advice given to some HIV positive mothers was incorrect and prevented EBF in those instances. This suggests that confusion still exists around HIV and infant feeding, because of the frequently changing guidelines.

Maternal-baby factors such as latching problems, baby rejecting the breast, fatigue from breastfeeding and perceived insufficiency of milk, have all been reported previously as key factors leading to early breastfeeding cessation [36, 37]. As in other studies, perceived insufficient milk supply was the most commonly reported maternal-baby issue among our participants. McCarter-Spaulding and Kearney’s [38] study indicated perceived insufficient milk at eight weeks postpartum was predictive of decreased EBF at 12 weeks. In their study, Hector et al., [39] maintain that only about 5% of women actually have physiological insufficient milk supply but up to 50% of mothers report to health facilities about insufficient milk for their baby [39]. Hill & Aldag explain this perception about lack of breast milk results from doubting one’s breastfeeding ability, which may lead to the perception of insufficient milk supply and adding other food or fluids to the baby’s diet [40].

Another important finding from this study was that, although there are over 12,000 CHW employed in KZN [41], and both the rural and urban clinics had CHWS deployed in the area, only two mothers reported being visited by a CHW. The role of CHWs includes visiting mothers in the post-partum period to provide counselling and support for infant feeding, and all CHWs receive training in infant feeding. CHWS are ideally situated to provide such support, which has been shown to lead to improved rates of EBF [42]. So it is concerning that mothers in this study were not visited by a CHW. However, we cannot generalize about CHW visits in other settings, as we only followed a small number of mothers. However, mothers could benefit from support at household level from CHW aimed at providing support and building confidence.

Family pressure to give the baby other food is cited as a factor influencing feeding choices in several African countries, including SA [26, 43–45]. A similar finding was reported in our study, where mothers were advised to add other food and traditional medicine to their babies’ diets. However, older mothers were less likely to succumb to family pressure compared to younger mothers. For mothers who had to adopt suboptimal feeding practices as an indication of obedience to family tradition, this meant diminished control on what the baby eats, and was a huge barrier to EBF for these mothers. This corroborates the findings by Kakute et al. who found that mothers sometimes introduce liquids or traditional medicine early, in keeping with traditional practice [46]. This implies that infant feeding interventions which focus on the mother without considering the mother’s position in the family and the culture of group decision-making, may fail in our context.

Consistent with the literature, maternal employment and schooling have been cited as key factors which may disrupt mothers’ plans to EBF [24]. For mothers who continued to exclusively breastfeed after returning to work or school, self-efficacy was the overarching enabler for such success. By its definition, self-efficacy drives one’s motivation [10]. In line with this definition, all the mothers who managed to exclusively breastfeed for six months also sought solutions such as expressing milk, stopping work and being with the baby all the time for the first six months. Mothers also had strategies in place, which they perceived as helping their breasts to produce more milk, which included drinking liquids such as tea and *amahewu* (liquid maize meal porridge).

Furthermore, this suggests self-efficacy enables mothers to seek solutions to enable breastfeeding even in the midst of challenges experienced. There is therefore an opportunity for the health system to adopt strategies aimed at enhancing mothers’ breastfeeding self-efficacy. Studies conducted in this area have found that strategies that aimed at increasing maternal self-efficacy instead of suggesting educational strategies, were more effective than those strategies that focus on enhancing knowledge [47, 48].

### Conclusion and implications for policy

Following mothers’ feeding practices prospectively provided a strong methodology for this study, and we were able to capture mothers’ feeding journeys thoroughly in the first six months. This study revealed that the health system is in a strong position to provide knowledge and support for mothers to make optimal feeding choices, increase mothers’ self-efficacy and thus increase EBF rates. According to Bandura’s theory of self-efficacy [37], social or verbal pressure can strengthen a person’s belief that they have what it takes to succeed [10]. In order to enhance self-efficacy, parents would need to be taught about challenges that may arise during early breastfeeding, so that they expect these and know how to overcome them. Additionally the importance of support by partners, other family or friends is important to build self-efficacy. However, despite additional support for breastfeeding, including deploying lay health workers to support breastfeeding in clinics and hospitals, a lot still needs to be done to build health workers knowledge and skills, to ensure that mothers receive appropriate messages and are able to overcome the challenges of EBF.

## Additional files

Additional file 1: KIBS Indepth Interview visit 2 Eng FINAL deployed. (PDF 280 kb)

Additional file 2: Indepth Interview V3-6 Eng FINAL deployed. (PDF 286 kb)

## Abbreviations

ANC: Antenatal Care
CHW: Community Health Workers
DoH: Department of Health
EBF: Exclusive Breastfeeding
IMCI: Integrated Management of Childhood Illnesses
KZN: KwaZulu-Natal
SA: South Africa
WHO: World Health Organization
