# Supplementary material for: Enablers and barriers to success among mothers planning to exclusively breastfeed for six months: a qualitative prospective cohort study in KwaZulu-Natal, South Africa
Source: Int Breastfeed J. 2017 Oct 3;12:43. doi: 10.1186/s13006-017-0135-8 (PMC5627494; doi:10.1186/s13006-017-0135-8)
Supplement: Supplementary file 1 — KIBS Indepth Interview visit 2 Eng FINAL deployed. (PDF 280 kb) [file 13006_2017_135_MOESM1_ESM.pdf]

KWAZULU-NATAL INITIATIVE FOR BREASTFEEDING SUPPORT:

In-depth interview guide Visit 2.

NB: *Before beginning the interview,* ***position*** *the microphone near to interviewer and interviewee,* ***turn it on*** *and* ***talk into the microphone*** *the following information:*

- *Area code*
- *Mother code*
- *Visit number (1-6)*

E.G. "This is (your name) I am conducting an interview in area 01 on mother 7 and it is visit number 3".

General:

1. How have things been going with feeding your baby?

Antenatal question:

1. When you were pregnant did anyone advise you on the feeding of your baby?

   Probe: who and what? In the clinic? Family or friends?

Post labour:

1. Can you tell me what happened directly after the baby was born?

   Probe

   a. *When did you first hold the baby?*
   b. *Did they put the baby on your chest?*
   c. *How soon after birth did you feed your baby?*

2. Describe the experience you had in starting to feed your baby in the hospital.
3. Was your baby given anything to drink BEFORE you started feeding him/her?
4. Did anyone help you with the feeding of your baby in the hospital?
   Probes:
   a. *Nurses/ lactation advisors*
   b. *Did you experience any challenges in feeding your baby while you were in the hospital?*
5. Did a lactation advisor (XXX name) help you with feeding your baby in the hospital?
6. When you got home did you experience any challenges in feeding your baby?
