# Supplementary material for: Enablers and barriers to success among mothers planning to exclusively breastfeed for six months: a qualitative prospective cohort study in KwaZulu-Natal, South Africa
Source: Int Breastfeed J. 2017 Oct 3;12:43. doi: 10.1186/s13006-017-0135-8 (PMC5627494; doi:10.1186/s13006-017-0135-8)
Supplement: Supplementary file 2 — Indepth Interview V3-6 Eng FINAL deployed. (PDF 286 kb) [file 13006_2017_135_MOESM2_ESM.pdf]

KWAZULU-NATAL INITIATIVE FOR BREASTFEEDING SUPPORT:

In-depth interview guide Visit 3-6.

NB: *Before beginning the interview,* ***position*** *the microphone near to interviewer and interviewee,* ***turn it on*** *and* ***talk into the microphone*** *the following information:*

- *Area code*
- *Mother code*
- *Visit number (1-6)*

E.G. "This is (your name) I am conducting an interview in area 01 on mother 7 and it is visit number 3".

Feeding patterns:

1. How have things been going with feeding your baby since we last visited?

   Probe:

   a. *Exclusive breast feeding/mixed feeding/formula feeding*
   b. *Have you given your baby any other fluids or formula milk? if so, why?*
   c. *Has the way you feed your baby changed since the baby was born? Explain.*

2. Why do you choose to feed your baby this way?
3. Have you had any challenges with feeding?

   Probes:

   a. *If so, what were those challenges?*
   b. *How did you resolve them?*

4. Who has given you advice on how to feed your baby? Discuss.

   Probes:
   a. *What advice have you been given?*
   b. *Do you think their advice affects you? Explain*
   c. *What have you decided after hearing such advice?*
   d. *How do you feel about others reactions to your decisions regarding feeding of your baby?*
   e. *Is there anyone who is supportive of your decisions?*
5. Who do you choose to listen to in terms of advice?
6. How do you feed your baby if you have to go somewhere without him/her?
   Probe:
   a. *Do you express milk/never leave her baby/ give her baby formula*

Exposure to health services:

7. Have you had a contact with the CCGs since you were pregnant?

   Probe:

   a. *How many times have you been visited?*
   b. *Have they helped you with feeding advice in any way?*

8. In your last clinic visit what advice did the staff give you regarding feeding your baby?

   Probe: Who was it that gave you that advice? Was it a nurse or nutritional advisor?

Social factors

9. What are the attitudes of family members and friends to the way you choose to feed your baby?

   Probe:

   a. *Who assists you financially*
   b. *Does the father have a say in how the baby is fed, whether its breastfeeding or formula?*
   c. *Ask about father's attitudes and paternal/maternal grandparents*
   d. *Have you experienced any negatives responses from people about the way you feed your baby?*

10. Is there any place where you would not feel comfortable feeding your baby?
11. Is there anything else I should know about the way you feed your baby?

ONLY FOR THE FINAL INTERVIEW

To conclude our visits, I want you to think back on how you fed your baby over the past 6 months. Can you describe that journey and the choices you made on the way?

Probe:

a. *Is there anything you would change or wish you had rather done regarding the feeding of your baby?*
b. *How do you think your baby's health has been over the six months? Explain*
c. *Do you think breastfeeding affected your health and your body in any way?*
